# Genome Sequence of Cluster BI1 Streptomyces griseus Phage TaidaOne

**DOI:** 10.1128/mra.00905-22

**Published:** 2022-10-31

**Authors:** Nai-Chun Lin, Chieh-Ling Liao, Che-Yu Cheng, Ping-Hua Chen, Shi-Wing Chen, Kuang-Chun Cheng, Marwin Fernandez, Ting-Kai Hou, Bo-Chen Jiang, Nai-Shun Liao, Tien Pao, Ying-Ying Wong, Hsiang-Yu Yang, Hui-Min Chung

**Affiliations:** a Department of Agricultural Chemistry, National Taiwan University, Taipei, Taiwan, Republic of China; b Department of Biology, San Francisco State University, San Francisco, California, USA; c Department of Plant Pathology and Microbiology, National Taiwan University, Taipei, Taiwan, Republic of China; d Master Program for Plant Medicine, National Taiwan University, Taipei, Taiwan, Republic of China; e Department of Biology, University of West Florida, Pensacola, Florida, USA; DOE Joint Genome Institute

## Abstract

Bacteriophage TaidaOne was isolated from soil collected in Taipei, Taiwan, using the host Streptomyces griseus. It is a siphovirus with a 56,183-bp genome that contains 86 protein-coding genes. Based on gene content similarity, it was assigned to actinobacteriophage subcluster BI1, within which only TaidaOne and GirlPower genomes contain an acetyltransferase homolog gene.

## ANNOUNCEMENT

**FIG 1:**
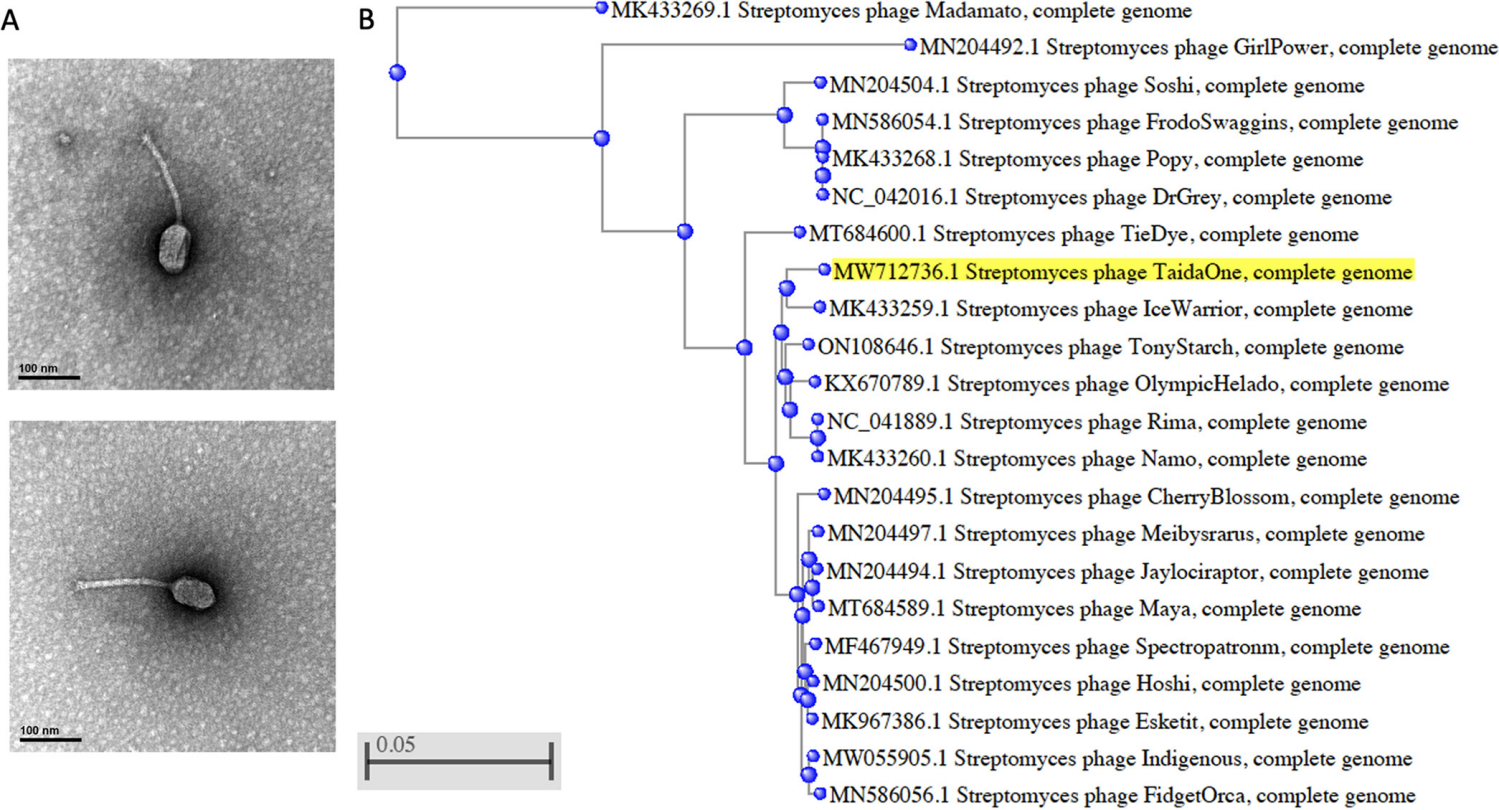
(A) Transmission electron microscopy of *Streptomyces* phage TaidaOne, showing it to be a siphovirus with a prolate capsid 75 to 80.7 nm in length and 45.8 to 50 nm in width and a flexible tail 158.3 to 163.5 nm in length (*n* = 2). A high-titer lysate (1 × 10^9^ PFU/mL) of TaidaOne placed on Formvar/carbon-coated copper grids was negatively stained with 1% uranyl acetate. (B) Phylogeny of BI1 phages. The graph was generated by BLAST using the neighbor-joining method, with a maximum difference of 0.75, to compute pairwise genome alignments between TaidaOne and the other 21 BI1 phages.

*Streptomyces* bacteria, which are well known for their production of antibiotics, are of great ecological and biomedical value (1, 2). The isolation and characterization of *Streptomyces* bacteriophages can help in the development of molecular tools to genetically manipulate *Streptomyces* strains (3–5). Here, we report on the isolation of a *Streptomyces* phage, TaidaOne, which was isolated from soil collected on 22 September 2019 from the campus of the National Taiwan University in Taipei, Taiwan (25.01849N, 121.542488E), using standard methods (6). The soil sample was washed with peptone-yeast-calcium (PYCa) liquid medium, and the wash was filtered through a 0.22-μm filter and inoculated with Streptomyces griseus (ATCC 10137). After incubation at 30°C for 3 days, the culture was filtered and the filtrate was plated in soft agar containing S. griseus (6), which resulted in the isolation of phage TaidaOne. TaidaOne was purified with three rounds of plating and forms clear plaques with a diameter of ~2 mm after 24 h at 30°C. Negative-staining transmission electron microscopy (7) revealed TaidaOne to possess a *Siphoviridae* morphology (Fig. 1A).

The TaidaOne DNA was isolated from a high-titer lysate and prepared for sequencing using the Norgen Biotek phage DNA isolation kit and the NEBNext Ultra II kit, respectively. Using an Illumina MiSeq system, 1,000,000 single-end 150-base reads were generated, which constituted 200-fold coverage of the genome. Raw reads were assembled using Newbler v2.9, and the resulting contig was checked for completeness and genome termini using Consed v29 (8), which revealed a genome of 56,183 bp, a G+C content of 59.5%, and 3′ single-stranded overhangs (5′-CGCCCGCCT-3′). TaidaOne was assigned to phage subcluster BI1 based on gene content similarity (GCS) of at least 35% to phages in the Actinobacteriophage Database (https://phagesdb.org) (9). The genome was annotated using DNA Master v5.23.6 (http://cobamide2.bio.pitt.edu), PECAAN (https://blog.kbrinsgd.org), Glimmer v3.02 (10), GeneMark v2.5 (11, 12), BLAST (13), HHpred (14), TMHMM v2.0 (15), TOPCONS v2 (16), ARAGORN v1.2.38 (17), tRNAscan-SE (18), and Phamerator (phamerator.org), all using default parameters. All genes are transcribed rightward. Eighty-six protein-coding genes were identified, 32 of which were assigned functions, including structure and assembly genes across the left half of the genome and DNA metabolism genes across the right half. An endolysin and a holin are encoded by genes 5 and 29, respectively. No immunity repressor or integrase functions could be identified, suggesting that TaidaOne is a lytic phage, consistent with other BI1 phages.

Among the 22 BI1 phages, a phylogenetic analysis performed using NCBI BLAST revealed TaidaOne to be most closely related to IceWarrior and then to Rima (KX670790), Namo (MK433260), OlympicHelado (KX670789), and TonyStarch (ON108646), with more than 98% nucleotide identity, and most distantly related to GirlPower and Madamato (Fig. 1B). The TaidaOne genome encodes a putative acetyltransferase that is absent in all BI1 phages except GirlPower.

### Data availability.

The sequence information for TaidaOne is available in GenBank with the accession number MW712736 and Sequence Read Archive (SRA) accession number SRX16259924.
